# History of attitudes toward death: a comparative study between Persian and western cultures

**Published:** 2016-12-26

**Authors:** Kiarash Aramesh

**Affiliations:** Associate Professor, Medical Ethics and History of Medicine Research Center, Tehran University of Medical Sciences, Tehran, Iran; Phd Candidate in Medical Ethics, Center for Healthcare Ethics, Duquesne University, Pittsburgh, PA, USA.

**Keywords:** *History of medicine*, *Death*, *Western culture*, *Persian culture*, *End of life*

## Abstract

In his seminal book on the historical periods of Western attitudes toward death, Philippe Aries describes four consecutive periods through which these attitudes evolved and transformed. According to him, the historical attitudes of Western cultures have passed through four major parts described above: “Tamed Death,” One’s Own Death,” “Thy Death,” and “Forbidden Death.” This paper, after exploring this concept through the lens of Persian Poetic Wisdom, concludes that he historical attitudes of Persian-speaking people toward death have generally passed through two major periods. The first period is an amalgamation of Aries’ “Tamed Death” and “One’s Own Death” periods, and the second period is an amalgamation of Aries’ “Thy Death” and “Forbidden Death” periods.

This paper explores the main differences and similarities of these two historical trends through a comparative review of the consecutive historical periods of attitudes toward death between the Western and Persian civilizations/cultures. Although both civilizations moved through broadly similar stages, some influential contextual factors have been very influential in shaping noteworthy differences between them. The concepts of after-death judgment and redemption/downfall dichotomy and practices like deathbed rituals and their evolution after enlightenment and modernity are almost common between the above two broad traditions. The chronology of events and some aspects of conceptual evolutions (such as the lack of the account of permanent death of nonbelievers in the Persian tradition) and ritualistic practices (such as the status of the tombs of Shiite Imams and the absolute lack of embalming and wake in the Persian/Shiite culture) are among the differences.

## Introduction

As an inseparable and inevitable part of human life, death has always been a source of reflection, imagination, and inspiration for thinkers, sages, artists, and even for ordinary people. The very questions of the nature of death and what happens after death have been answered in many deferent ways and forms by religions, philosophies, and traditions. These answers along with the reflections, imaginations, and emotions shaped attitudes surrounding the concept and reality of death, and these attitudes have been embodied and symbolized in the death-related rituals and typical behaviors from the times close to one’s death until the funeral, burial, and memorial ceremonies. These thoughts, imaginations, behaviors, and rituals, and in short, these attitudes have shaped their own history throughout the ages (1).

Western and Persian civilizations are two old and large civilizations with their own unique characteristics and attitudes toward existential subjects such as death. It has been argued the speaking of the “Western attitude” contains a sort of overgeneralization and ignoring the diversity, which exists among the Western cultures (2). However, as showed in the seminal work of Philippe Aries, which is discussed below, one can find general trends in culture and mentalities in Western civilization with a valid methodology and defendable results (1). The same can be said about the Persian culture.

As described below, in the Western history, thoughts and attitudes toward death have been under the influence of their Greek/Latin roots and then by Christianity, Medieval schools of thought, Enlightenment, and Modernity. The Persian attitudes, however, have been under the influence of Zoroastrianism (Ancient Persian religion which was the official religion of the Sassanid Empire before the Islamic era), Islam and its Shi’a denomination, Sufism, and Modernity. The gist of these attitudes has been mirrored in the Persian Poetic Wisdom. The Persian Poetic Wisdom is a large body of Persian poems carrying the collective wisdom and accumulated historical experiences of Persian-speaking people with a specific role in transferring culture and morality through generations. In this paper, the most prominent parts of this historical collection have been used for exploring the attitudes of Persian people throughout the history.

In this paper, through a comparative review of the consecutive historical periods of attitudes toward death between the Western and Persian civilizations/cultures, the main differences and similarities of these two historical trends are explored. It has been showed that although both civilizations moved through broadly similar stages, some influential contextual factors have been very influential in shaping noteworthy differences between them.

**Historical Periods**

Philippe Aries, the French author and thinker, in his seminal book titled Western Attitudes toward Death: From the Middle Ages to the Present has discussed the historical periods of Western attitudes toward death. This book has been one of the most influential intellectual works about the history of attitudes toward death in the West and has been applauded by many of critics and scholars. Philippe Aries describes four consecutive periods through which these attitudes evolved and transformed (1). For this purpose, Aries uses literature, such as ancient texts, romances, and novels to explore and retrieve the attitudes of people at each age/period he studying. This methodology has been criticized (2). However, it seems to be the best available method to dig the history and find the beliefs and attitudes of people who died hundreds years ago.

Rooted in the Persian Empire established in the 6^th^ century BCE, the Persian culture has mover through a turbulent historical route. The first official religion of the Persian Culture was Zoroastrianism, which replaced by Islam after the invasion of Arabs in the beginning of the 7^th^ century. Consequently, the majority of Persians adopted the Shi’a (also called Shiite) domination of Islam by around 16^th^ century. Nowadays the majority of people in Iran, Tajikistan, and Afghanistan speak Persian also called Farsi). Persian-speaking people share a common cultural history and heritage. The most important element of this common cultural heritage is the Persian Poetic Wisdom. In this paper, the focus of the study was on the history of Iran as the major part and homeland of Persian culture. In the medieval Persian literature, in the absence of novels and scarcity of long non-poetic romantic narrations, poetry holds almost all the functions of literature for Persian-speaking populations. By exploring the Persian Poetic Wisdom, one can retrieve the Persian attitudes toward death it the historical evolutions of these attitudes.

Below, using the findings of Aries and the main sources of the Persian Poetic Wisdom, each period is briefly described based on Aries’ account of Western mentalities and compared with its counterpart in the Persian history.

*Tamed death*

According to Aries, this period is characterized by regarding death as an integral part of daily life. The typical picture of dying person is a person laying in his/her deathbed, aware of his/her imminent death and welcoming to it, with his/her family members and friend and neighbors surrounding him, and the priest coming upon the call of the dying person when he/she feels that it is the time, and accepting the death of loved ones without emotional burst and impatience, but with calm and acceptance. In this typical picture, death is witnessed by friends and relatives, even young children (1). The picture of a deathbed surrounded by friends and family members is also a common and familiar picture in the pieces of the Persian Poetic Wisdom in the medieval era (3).

According to Aries, the burial places are away from homes and towns and usually are located in the churchyards. In the churchyards, the dead are under the protection of the saints/church and will resurrect at the time of the second coming of/Jesus. The nonbelievers simply wont resurrect. They will remain dead (1). In the Persian culture and the Persian Poetic Wisdom, although, some Sufi thinkers implicitly mentioned that some people wont be resurrected in the afterlife because of their devolution to this world’s pleasures and not noticing spiritual messages, they still believed in the day of judgment and personal responsibility toward ones deeds in the afterlife (4). According to Aries/portrayal, churchyards were also places for recreational gatherings of people. Graves are not expected to be permanent, and ossuaries are common (1). This period ends around the 12^th^ century.

In the ancient Persia, before Islam, the official religion, Zoroastrianism, was greatly influential on the lifestyle and beliefs of people regarding existential issues including death and its related rituals. For protecting the soil, as a pure element, from contamination, Zoroastrians did not bury the dead bodies, instead, the left them in special places, namely *Dakhmeh*, to be decomposed by natural forces and eaten by wild animals (5). This kind of ritual is similar to the burial rituals of the tamed death era as described by Aries, in not keeping permanent and personal and decorated tombs.

The Persian religions, however, from the very beginning, either Zoroastrianism or Islam advocated the God/Devil dichotomy which is translated to redemption/downfall dichotomy in the afterlife (6).

*One’s own death*

In the period starting around the 12^th^ century, namely the second Middle Ages, through the gradual and subtle changes of the vulgate, the “personalization of death” takes place. The teachings of the Christian church contending that every person will be judged after their deaths and will be rewarded of punished according to their deeds reshaped the Western attitudes toward death. Therefore, death became more personal. Consequently, the ceremonial meaning of deathbed changed from witnessing death to witnessing the moments before judgment in which the behaviors of dying person implies his/her redemption or downfall. In addition, having personal and personalized encrypted tombs became common (1). In this period, the second coming of Jesus is considered as the time of redemption for believers and downfall for nonbelievers and sinners; therefore, death is no longer a common and same fate and destiny of all people, but it is the time of salvation for some and devastation for others (1).

This period shows the main characteristics of the Persian attitudes toward death from the medieval ages until the modern era. The mainstream religious part of the Persian culture firmly believes in the Day of Judgment. The graveyards and cemeteries are outside the cities. One major difference in this regard appeared after dominance of Shiite domination in Persian-speaking territories. According to Sunni Islam, decoration tombs and having ceremonies and memorials in the graveyards is forbidden. It is strictly prohibited for women to visit tombs and graveyards (7). In Shiite Islam, in contrast, visiting and praying in the tombs of innocent Imams is part of very emphasized religious rituals. Therefore, paying attention to decorating and ornamenting tombs became popular after Shiite denomination became the official religion in *Safavid* Iran from the beginning of the 16^th^ century. Graveyards and cemeteries grown and expanded around the tombs of innocent Imams and their offspring (*Imamzadeh*) and became a place for religious and social events and gatherings of people. In medieval towns of Iran, tombs and cemeteries were in close distance to the town and people visited them frequently. The dream of a religious Shiite common man was (and still is) having a pilgrimage to the tomb of an innocent Imam (Specially Imam Hossein in Karbala, Iraq; Imam Ali in Najaf, Iraq; and Imam Reza in Mashhad, Iran). Some Imams are buried in Sunni countries, such as the Saudi Arabia and Shiites, cannot build tombs for them and visit their tombs.

Rituals of deathbed in this period are very similar to Arias/description of the first and second periods of Western attitudes. In the most seminal and famous book of moral teachings in Persian literature, namely Golestan (means the garden) that is a major part of the Persian Poetic Wisdom, Musleheddin Saadi narrates many tales of life advices given by old and wise people to their sons or friends in their deathbeds (3). The pictures of deathbed portrayed by Saadi are very similar to the ones depicted in medieval literature of Western culture (1).

One of the major aspects of similarity between the Western and Persian medieval cultures is the religious-based belief in the sanctity of life (8). This idea is originated in Christianity and Islam/Sufism in Western and Persian cultures, respectively (9). Stress on giving the utmost values to preserving innocent lives along with condemning killing ones self or others is part of the beliefs surrounding the concept of death in both traditions.

*Thy death*

Beginning from early 18^th^ century, this period, according to Aries is characterized by replacing acceptance with fear and witnessing death with mourning for the dead. In this period, death, like sex, is not considered a normal and pretty familiar part of the reality of human’s life, but it is exalted and feared. In this new picture, families and friends around the deathbed are not witnessing death (or the moment before judgment); they are mourning. This mourning is accompanied by expressing excessive emotions showing intolerance of the death of loved one. This heightened and vivid expression of emotions makes this period a “sentimental era.” This emotional and dramatic mourning continues with preserving the memory of the lost loved ones. Consequently, tombs become more ornate and elaborate especially in Southern Catholic countries of the Western Europe (1).

In the Persian speaking countries, the enlightenment theories imported at around the last decades of the 19^th^ century. It seems that exalted expression of emotions is still part of burial and memorial ceremonies though deeply religious people try to refrain from showing excessive emotions while mourning. At this time, new forms of expression were introduced to the Persian literature and under the influence of modern Western culture and literature, the first novels were created in Persian, followed by theatrical pieces and movies (10).

*Forbidden death*

Beginning in the late 19^th^ and early 20^th^ centuries, the last period of western attitudes toward death, according to Philippe Ares, is demarcated by sex being replaced by death as a taboo in Western cultures. The establishment and popularity of the institution of hospital, along with modern and sophisticated health-care technologies, transformed the typical picture of death from “a predicted/expected death in one’s own home and surrounded by friends and family” to “unwanted and fought against death on the hospital bed, while one is unconscious, alone, and tried to eschew death until the last minutes.” In this era, the exalted emotions of the previous period are replaced by ignorance toward death and trying to eliminate it from the picture of normal and daily life. Practices such as cremation, embalming and wake, and commercialization of funerals in the form of emergence of funeral directors who market their services and products as “consumer goods” are among the manifestations of this last transformation of the Western attitudes toward death (1).

Persian-speaking societies encountered modernity as a series of imported ideas from the west. They became familiar with the modernized west and the ideas and theories of enlightenment, French revolution, and modernity in the last decades of the 19^th^ century. This acquaintance was accompanied by awareness of their historical backwardness. Although some parts of the community tried to adopt the attitudes and lifestyles compatible by modernity, other parts of society were reluctant to accept it because of religious and cultural hesitations. The resulted conflicts have not been solved in these societies yet. Actually, the Pahlavi Dynasty in the 20^th^ century tried to modernize Iran and import and advocate different aspects of modern and Western life. The 1979 revolution, which ended up in empowering the anti-western Shiite clergies, was a major setback of the movement toward Western modernization. Persian attitudes toward death and the death-related rituals, as a cultural construct, has been largely under the influence of these cultural transformations and conflicts. Nowadays, Persian-speaking societies encompass an amalgamation of very different lifestyles and cultural attitudes.

Decorating tombs and showing the wealth and social status of family in the funeral and burial and memorial ceremonies has been more common. As described above, treating death as “consumer good” is also part of the last historical period of attitudes toward death. Growth of funeral industry and their marketing effort is a well-known phenomenon in the United States. The similar phenomenon can be witnessed in Iran. Advertisements for different parts of funeral and burial rituals can be seen now and then. For example, advertisements for filming, food delivery, and artworks on tombstones and also ritual singing (*Maddahi*) at the funeral and memorial ceremonies are part of this highly profitable market.

In this era, some intellectuals started a kind of re-reading and re-interpreting of the major pieces of the Persian Poetic Wisdom to criticize the traditional religious customs, conventions, and rituals. Referring to Hafez and Khayyam, two of most famous Persian poets became very popular. Their poems were cited to emphasize a teaching, which is one of the core cultural beliefs if this period according to Aries: The centrality of happiness in this life and recalling the inevitability and brutality of death for emphasizing on seeking happiness in the worldly life.

Because of the influence of Shiite Islam, practices like embalming and wake have never been practiced in Persian speaking societies. These practices are strictly forbidden in Shiite Islam. According to the Islamic teachings, the dead body must be buried as soon as possible and preserving the dead body is strictly prohibited (11). There are just a few exceptions: Such as mummifying the dead body of Reza Shah, the first king of the Pahlavi Dynasty who died in exile and has to transferred to his permanent tomb in Iran and recent permissions of preserving bodies for teaching anatomy in medical schools, of course with emphasis of the priority of using the bodies of non-Muslims.

## Conclusion

In this paper, for a brief comparison of the Western and Persian attitudes toward death, two main resources were used: The seminal work of Philippe Aries on the Western attitudes, and the Persian Poetic Wisdom and author’s description of the contemporary Iran along with historical resources for exploring the Persian attitudes. As described above, the historical attitudes of Western cultures have passed through four major parts described above: “Tamed Death,” “One’s Own Death,” “Thy Death,” and “Forbidden Death.” The historical attitudes of Persian-speaking people toward death have generally passed through two major periods. The first period is an amalgamation of Aries’ “Tamed Death” and “One’s Own Death” periods and the second period is an amalgamation of Aries’ “Thy Death” and “Forbidden Death” periods.

Through this historical comparative study, one can find the main similarities and differences of the historical evolutions of attitudes toward death between these two cultures/civilizations. The concepts of after-death judgment and redemption/downfall dichotomy, and practices like deathbed rituals and their evolution after enlightenment and modernity are almost common between the above two broad traditions. The chronology of events and some aspects of conceptual evolutions (such as the lack of the account of permanent death of nonbelievers in the Persian tradition) and ritualistic practices (such as the status of the tombs of Shiite Imams and the absolute lack of embalming and wake in the Persian/Shiite culture are among the differences.

The above-mentioned similarities show that one can find similar general trends even in difference cultures. Studying these similarities can pace the way for constructing and advocating common cultural norms and principles in the era of globalization.
